# Beyond Janus Geometry: Characterization of Flow Fields around Nonspherical Photocatalytic Microswimmers

**DOI:** 10.1002/advs.202105009

**Published:** 2022-07-15

**Authors:** Sandra Heckel, Clemens Bilsing, Martin Wittmann, Thomas Gemming, Lars Büttner, Jürgen Czarske, Juliane Simmchen

**Affiliations:** ^1^ TU Dresden Chair of Physical Chemistry Zellescher Weg 19 01069 Dresden Germany; ^2^ TU Dresden Laboratory for Measurement and Sensor System Technique Helmholtzstraße 18 01069 Dresden Germany; ^3^ Leibniz Institute for Solid State and Materials Research Dresden Helmholtzstraße 20 01069 Dresden Germany; ^4^ Competence Center Biomedical Computational Laser Systms (BIOLAS) Helmholtzstraße 18 01069 Dresden Germany

**Keywords:** flow fields, microswimmers, particle tracking velocimetry, photocatalysis

## Abstract

Catalytic microswimmers that move by a phoretic mechanism in response to a self‐induced chemical gradient are often obtained by the design of spherical janus microparticles, which suffer from multi‐step fabrication and low yields. Approaches that circumvent laborious multi‐step fabrication include the exploitation of the possibility of nonuniform catalytic activity along the surface of irregular particle shapes, local excitation or intrinsic asymmetry. Unfortunately, the effects on the generation of motion remain poorly understood. In this work, single crystalline BiVO_4_ microswimmers are presented that rely on a strict inherent asymmetry of charge‐carrier distribution under illumination. The origin of the asymmetrical flow pattern is elucidated because of the high spatial resolution of measured flow fields around pinned BiVO_4_ colloids. As a result the flow from oxidative to reductive particle sides is confirmed. Distribution of oxidation and reduction reactions suggests a dominant self‐electrophoretic motion mechanism with a source quadrupole as the origin of the induced flows. It is shown that the symmetry of the flow fields is broken by self‐shadowing of the particles and synthetic surface defects that impact the photocatalytic activity of the microswimmers. The results demonstrate the complexity of symmetry breaking in nonspherical microswimmers and emphasize the role of self‐shadowing for photocatalytic microswimmers. The findings are leading the way toward understanding of propulsion mechanisms of phoretic colloids of various shapes.

## Graphical abstract

BiVO_4_ single crystal microswimmers swim through a degradation of peroxide without a specific asymmetrization step. Particle tracking velocimetry gives access to the flow pattern enabling the motion.

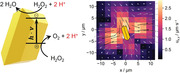

## 1 Introduction

The design of artificial active matter, that is, particles that self‐propel in a certain direction, is a challenging yet promising subject with envisioned applications in a range of fields like environmental remediation,^[^ ^1^, ^2^, ^3^ ^]^ sensing,^[^ ^4^ ^]^ and biomedical engineering.^[^ ^5^, ^6^, ^7^ ^]^ Due to the physical laws for fluid flow on the microscale, constant reaction throughput that dissipates energy and reaches out‐of‐equilibrium conditions is required to induce active motion.^[^ ^8^ ^]^ A synthetic realization are catalytic microswimmers, which typically drive catalytic peroxide degradation on their surface and consequently are understood to move by an interplay of self‐diffusiophoretic and self‐electrophoretic forces, depending on the specific morphology and reactions taking place.^[^ ^9^, ^10^, ^11^ ^]^ In a nutshell, interactions between the solute, the swimmer surface, and the substrate on which they are moving cause fluid flows around the colloids and subsequently active motion. In order for a preferred motion direction to arise, the catalytic reaction needs to take place asymmetrically over the surface of the microswimmer, which is often achieved by the fabrication of janus particles.^[^ ^12^, ^13^ ^]^ The understanding of the emerging flows around microswimmers is essential to elucidate their motion behavior, mechanism, and interactions between active and passive colloids. Theoretically, potential flow fields can be calculated as solutions of the Navier–Stokes equations.^[^ ^14^ ^]^ If incompressible flow is assumed, that is, fluid motion is induced by solute‐surface interactions, force multipoles that result in pusher or puller‐type swimmers are solutions of the equations. Furthermore, the chemical reactions taking place on the surface of a catalytic microswimmer may give rise to source multipoles or neutral microswimmers, where the fluid motion is determined by chemical flows.^[^ ^14^, ^15^ ^]^ Experimental characterization of self‐induced flow fields around microobjects is challenging, and only few examples exist.^[^ ^16^, ^17^, ^18^ ^]^ For catalytic polystyrene‐platinum (Pt@PS) janus spheres, which have been a frequently used microswimmer model system,^[^ ^10^, ^11^, ^19^, ^20^, ^21^ ^]^ Campbell et al. determined pusher‐type flow fields with current loops from the equator to the platinum pole of the particles.^[^ ^16^ ^]^ However, despite the high reproducibility and shape uniformity at which spherical particles can be synthesized, their transformation into janus particles represents the bottleneck of this process. As metallic catalysts are commonly deposited in a separate step, often by thermal,^[^ ^22^ ^]^ sputter,^[^ ^23^ ^]^ or electron‐beam^[^ ^24^ ^]^ deposition, the preparation of particle monolayers is required. This strongly decreases the yield of catalytic janus particles that can be obtained in one batch. As an alternative, single‐component microswimmers that contain an inherent source of asymmetry such as their shape are being investigated.^[^ ^25^, ^26^ ^]^ This rises the question about the corresponding flow conditions around these microswimmers. Despite a variety of experimental shapes for active matter, modeling beyond spherical or spheroidal geometries has barely been approached. Recently, we and others proposed photocatalytic single‐component BiVO_4_ microswimmers that rely on charge‐carrier separation due to a surface heterojunction between catalytically active crystal facets.^[^ ^27^, ^28^ ^]^ In different studies, BiVO_4_ colloids^[^ ^29^ ^]^ have shown great potential for the investigation of the behavior of single microswimmers,^[^ ^30^ ^]^ but also active assemblies and interaction with passive particles have been demonstrated.^[^ ^31^, ^32^ ^]^ Considering that for such microswimmers neither the prediction of expected flows by established theoretical models, nor their experimental resolution has been approached, the understanding of the rise of active motion through facet selective oxidation and reduction reactions lags behind their well‐known janus‐shaped counterparts.

Here, we develop an experimentally viable approach to determine flow fields around pinned single‐crystalline BiVO_4_ microswimmers. By that, we aim at gaining insight into the motion mechanism of photocatalytic, nonspherically shaped microparticles, both of which are properties that make for a challenging experimental realization but are capable of contributing greatly to the understanding of the fundamental processes behind active micromotion. In order to obtain comparable flow patterns, we first optimize the synthesis of these particles with shape‐directing surfactants. By particle tracking velocimetry of passive gold tracer particles around pinned microswimmers, we are able to resolve flow patterns around the swimmers and relate these to the observed behavior under illumination. By averaging flow fields of individual particles, we are able to distinguish common trends from effects related to individual particle morphology.

## 2 Characterization of BiVO_4_ Microparticles

**Figure 1:**
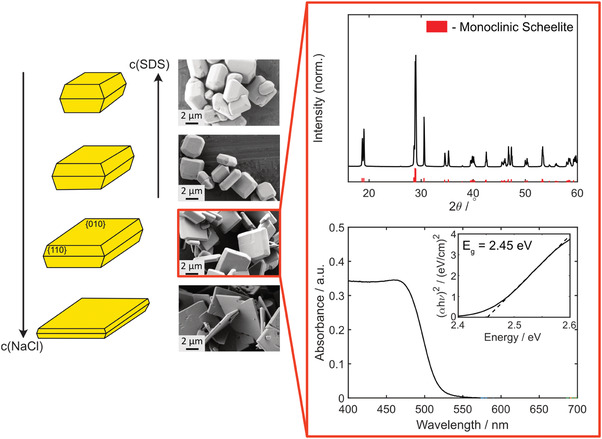
Left: Scheme showing the impact of increasing concentration of NaCl and SDS marked by arrows on crystal morphology and SEM images of BiVO_4_ particles synthesized at different concentrations of NaCl and SDS: top: 0.05 m NaCl and 0.05 MSDS; second: 0.05 m NaCl and 0.005 m SDS; third: 0.05 m NaCl; bottom: 1.0 m NaCl. Right: XRD pattern and UV–vis absorption plot of the selected BiVO_4_ sample. The monoclinic scheelite crystal structure can be clearly identified (Reference: PDF‐Nr. 16‐688, red). In the absorption spectrum, which was recorded with a dilute particle solution in water, it can be ssen that light in the UV up to the visible blue range is absorbed. Calculation of the band gap via the Tauc plot for a direct semiconductor gives a value of *E*_g_ = 2.45eV.

BiVO_4_ has been demonstrated in different morphologies from undefined crystals^[^ ^33^ ^]^ to shuriken,^[^ ^30^, ^32^ ^]^ and spheroids.^[^ ^31^ ^]^ While for bulk chemical reactions the morphologies of individual colloids is of less importance, a low polydispersity is crucial to ensure comparable hydrdynamic conditions. To achieve homogeneous particle dimensions, as a first step the synthesis of BiVO_4_ was optimized. Synthesis was carried out using a hydrothermal reaction, where the morphology of the resulting crystallites can be controlled by addition of a capping agent.^[^ ^34^ ^]^ A well known capping agent for BiVO_4_ are Cl^−^ ions, which selectively adsorb onto the {010} facets and reduce the surface energy. Consequently, growth of these facets is slowed down, which means that an increase in Cl^−^ concentration by addition of NaCl results in larger exposed {010} facets.^[^ ^35^ ^]^ It was also reported, that sodium dodecyl sulfate (SDS) is selectively adsorbed onto {110} facets of BiVO_4_, which qualifies it as a capping agent for those facets.^[^ ^36^ ^]^ In this work, the combination of different NaCl and SDS concentrations was investigated to obtain homogeneous particle sizes and shapes within one synthesis batch. The obtained results are illustrated in scanning electron microscopy (SEM) images in the left panel of **Figure** **1**. As can be seen, four different samples with varying NaCl and SDS concentrations have been studied. As expected, particles that formed in the presence of only NaCl are characterized by preferentially exposed {010} facets. Additionally, it can be seen that this also leads to a decrease in colloid thickness, which is accompanied by a tendency for breaking in the sample with the highest NaCl concentration (1.0 m). When SDS is added to the synthesis, the microparticles appear to become thicker and smaller, which can be attributed to the surface capping of {110} facets. Although breaking is not an issue with these colloids anymore, the smaller the microswimmers are, the more challenging it becomes to resolve fluid flow patterns in their vicinity. For the desired flow field investigation, particles synthesized with 0.05 m NaCl only therefore appear to be the best candidates, having a comparably high edge length of 4.2±1.0 µm and a thickness of 0.84±0.34 µm. They were therefore chosen for the following experiments. Additional analysis yielding absorption spectra and X‐ray diffractograms (XRD) confirm the monoclinic crystal structure and a band gap of 2.45 eV (Figure 1, right panel), which confirms the applicability of the BiVO_4_ colloids as photocatalytic microswimmers under ultraviolet (UV) illumination. In the further course of this manuscript, all experiments were carried out with the BiVO_4_ colloids from this synthesis batch.

**Figure 2:**
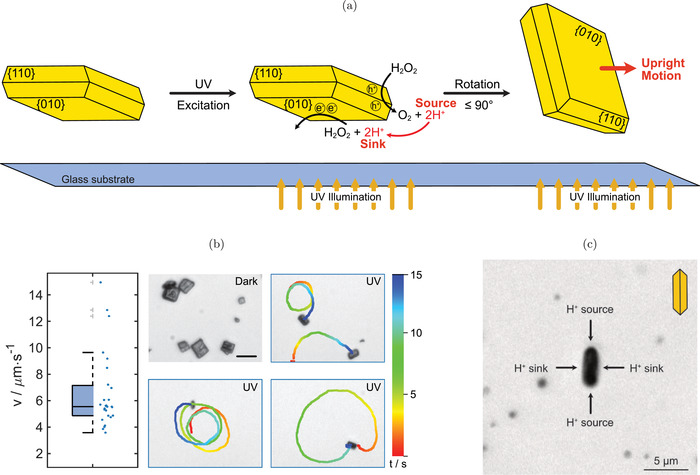
a) Scheme illustrating the excitation process of the BiVO_4_ single crystals. UV (385 nm) excitation from the bottom induces the separation of excited electrons onto {010} facets and holes onto {110} facets. In consequence, spatially separated oxidation and reduction reactions of H_2_O_2_ lead to a proton flow from {110} to {010} facets on the colloid surface. The particles rotate by up to 90° in reference to the glass substrate and then move parallel to the substrate in the direction of the reductive facet, which we call upright motion. The distance between the particle and the glass substrate is not to scale. b) Speed box plot of single crystalline BiVO_4_ microswimmers under UV illumination in dilute H_2_O_2_. Solid horizontal line shows median, dashed horizontal line refers to mean. Gray markers refer to outliers, whiskers indicate interquartile range. The blue scatter shows the individual speed values from which the box plot is created. On the right, microscope snapshots show particles in dark condition, where it can be seen that they lay flat on their {010} facet. Under illumination, upright motion with a tendency for circular trajectories is observed. Scale bar is 10µm. c) Illustration of a typical snapshot of a microswimmer particle under illumination during flow field acquirement. The particle is pinned to the substrate in a 90° angle, as the scheme in the top right indicates. It is surrounded by gold tracer particles. Due to the truncated bipyramidal morphology of the microswimmer, two reductive facets ({010}, H^+^ sinks) and two oxidative facets ({110}, H^+^ source) are exposed to UV excitation in the substrate plane where the flow field is determined.

The active motion of the microparticles was investigated by speed and mean‐squared displacement (MSD) analysis under UV illumination (385 nm wavelength) in 0.1 wt% H_2_O_2_ solution. The results are illustrated in **Figure** **2**. The processes taking place on the particle surface under these conditions are schematically shown in Figure 2a. When no UV excitation is present, the particles lay flat on their {010} facets on the glass substrate. It should be noted that the colloids neither show any active motion in these dark, illumination‐free conditions, nor under illumination in absence of H_2_O_2_, which is an informative indicator that the importance of thermophoretic motion is minimal, most likely because the fraction of light that contributes to heating is very small. The diffusion constant of their Brownian motion under these conditions was determined as 0.19±0.01 and 0.24±0.004 µm^2^ s^−1^, respectively.

When UV excitation is turned on, the colloids are illuminated from the bottom. Under these conditions, H_2_O_2_ is decomposed into water and oxygen in two separate half‐reactions: Due to charge‐carrier separation on the BiVO_4_ surface, this reaction is assumed to be split into an oxidation on {110} facets and a reduction on {010} facets. This has also been shown by photodeposition experiments (see Figure S1, Supporting Information).^[^ ^33^ ^]^ Oxidative facets on the particle sides now act as proton sources, while reductive facets consume protons. Interestingly, we observed a tendency for the particles to reorient themselves under illumination: While laying on {010} facets under dark conditions, the colloids rotate in respect to the substrate under UV excitation by an angle α of up to 90°, which results in an upright position (see Video S1, Supporting Information). Once reoriented, the microswimmers move parallel to the substrate in a direction perpendicular to their reductive facets with a tendency for curved trajectories. This is in agreement with observations of previously studied BiVO_4_ microswimmers with comparable shapes.^[^ ^30^ ^]^ The mean speed of individual BiVO_4_ microswimmers as a data scatter/box plot as well as examples for particle trajectories are shown in Figure 2b. Due to the microparticle geometry, curved trajectories can be expected from nonaxisymmetric microswimmers as they possess an intrinsic angular velocity.^[^ ^14^ ^]^ A mean speed of 6.59±2.89 µm s^−1^ can be observed. Figure 2b also illustrates that mean speeds as well as trajectory curvatures are scattered over a wide range of values. This can be explained by the polydispersity of the microparticles within one synthesis batch, which even after optimization is still higher than what is known for spherical janus microswimmers.^[^ ^22^ ^]^ Yet, it should be pointed out that despite the size differences, all of the BiVO_4_ microswimmers appear to show the upright rotation upon illumination and a tendency for curved tajectories. Analysis of the MSD of an exemplary particle further confirms the active motion of the microswimmers (see Figure S2, Supporting Information). Compared to earlier presented morphologies, the activity and speed of these microswimmers is strongly increased, which can be justified by the higher catalytic activity of the single crystalline structures.^[^ ^30^, ^31^, ^32^ ^]^ The observed rotation into an upright position is likely caused by unidirectional illumination from the bottom of the sample, which initially provokes a catalytic activity on the bottom reductive {010} facet on which the particles are laying when in passive Brownian motion. The thereby caused osmotic pressure could induce an upright rotation.^[^ ^37^ ^]^ Fixed swimming positions are also observed in other catalytic microswimmers, where a complex interplay of interactions has been found responsible.^[^ ^38^ ^]^ The precise swimming mode of these and other BiVO_4_ microswimmers also depends on environmental conditions such as the pH of the solvent, as we studied in more detail in recent publications.^[^ ^30^, ^39^ ^]^ In future studies, we envision to gain deeper understanding of this behavior with the help of simulations of the behavior of these uniquely shaped microswimmers.

## 3 Flow Field Analysis

Prior to flow field analysis, the motion mechanism and resulting expectations for the fluid flow shall be considered. The strong separation of reduction and oxidation reaction on the surface of these colloids suggests a dominant self‐electrophoretic motion mechanism. This hypothesis is supported by previous works on polycrystalline BiVO_4_ microswimmers, for which a similar spatial separation of oxidative and reductive particle faces was shown. For these particles, decreasing speeds upon increase of the salt concentration (NaCl) are an additional indicator for a self‐electrophoretic motion mechanism.^[^ ^39^ ^]^ For the well‐known example of bimetallic self‐electrophoretic rods that also move by H_2_O_2_ decomposition, fluid flows from oxidation to reduction site were predicted, which is in agreement with a source dipole field.^[^ ^40^ ^]^ If these considerations are transferred to the single crystalline BiVO_4_ particles under illumination, proton flows from {110} to {010} facets can be expected. Due to the upright orientation of microswimmers under illumination, two proton sources and two proton sinks are anticipated in the substrate plane where motion is observed, causing a source quadrupole (see Figure 2c). However, active motion can only be observed if the interactions between the solute and the microswimmer surface are nonuniform across this surface, which then causes an asymmetric flow field. In janus particles or bimetallic rods, this is caused by different interaction potentials of the individual, chemically different, hemispheres with the solutes.^[^ ^9^ ^]^ The particles discussed here only consist of a single component, namely BiVO_4_. Due to the monoclinic crystal structure, different atoms are exposed on the different facets, which could cause a dependence of the surface‐solute interaction.^[^ ^41^ ^]^ Yet, for the truncated bipyramidal particle morphology, this should still result in equivalent solute‐surface interactions for crystallographically identical facets and eventually a symmetric flow field due to the mirror symmetry of the particles. However, synthetic surface defects were identified in SEM images, which render the photocatalytic activity of each particle side unique (see Figure S3, Supporting Information). Furthermore, the illumination direction of the samples needs to be considered. In the conducted microscopy experiments, samples were illuminated from the bottom through the objective with a UV LED. Due to the high absorption coefficient of BiVO_4_ in the UV range (see Figure S4, Supporting Information), it can be assumed that particle sides which are oriented toward the substrate receive more light excitation and therefore have a higher photocatalytic activity than particles sides which are oriented toward the top of the sample.^[^ ^42^ ^]^ In consequence, these two effects are considered as the main cause for nonuniform, asymmetric BiVO_4_ surface‐solute interactions in spite of a mirrorsymmetric particle morphology. We therefore assume the self‐electrophoretic motion mechanism to be induced by charge‐carrier separation onto different facets which leads to proton flows and oxygen gradients under illumination.

**Figure 3:**
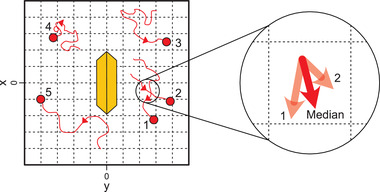
Raster model of PTV processing. In each raster point, the median flow speed and direction is calculated from all tracer particle trajectories present.

Flow fields were obtained by particle tracking velocimetry (PTV). To ensure good visibility in optical microscopy despite small particle sizes, gold nanoparticles were synthesized as passive tracers, which can be tracked to reconstruct the fluid flow without modifying it significantly themselves. The quasispherical nanoparticles have a mean diameter of 250±22 nm and did not show any catalytic activity toward H_2_O_2_ decomposition (see Figure S5, Supporting Information). Due to citrate ligands present on their surface, the gold tracers appear to have a slightly negative surface charge. This needs to be kept in mind for their motion analysis afterward, as it will mainly be influenced by the fluid flow, but to a lesser extent likely also by electrophoretic motion of the tracers in the electric field of our microswimmers.^[^ ^43^ ^]^ For video recording, solutions of 0.2 wt% H_2_O_2_ were prepared and BiVO_4_ microswimmers as well as Au tracer particles were added. The samples were applied to a glass substrate and subsequently covered with a thin cover glass and sealed to prevent evaporation. Under these conditions, the colloids were confined in samples with a height of *h* ≈ 30µm. To intensify fluid flows, microswimmers were pinned to the glass substrates using high light intensities in upright (α = 90°) or inclined (0° < α < 90°) position which causes them to act as fluid pumps. For video recording, the substrates were inserted in an inverted microscope and illuminated with UV light from the bottom. In PTV analysis, the median speed and motion direction of the tracers was determined for a rasterized display of the surrounding area of the pinned microswimmers (see **Figure** **3**). Details on the implementation can be found in Section 5. The tracer motion can also be seen in Video S2, Supporting Information.

**Figure 4:**
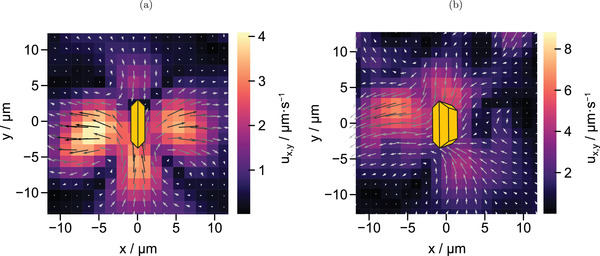
Flow fields of exemplary particles which are oriented in a) upright and b) inclined position. Flow direction is shown by arrows and velocity is indicated by the color map with $u_{x,y}=\sqrt{u_{x}^{2}+u_{y}^{2}}$. Inward flows toward oxidative {110} facets and outward flows from reductive {010} facets can be identified for both particles. Note that these fields were obtained for pinned microswimmers, which is why they do not contain an indication of the motion direction. However, as was indicated in motion studies, the microswimmers generally move parallel to the substrate in a direction perpendicular to their reductive facets, which would be along the *x*‐axis of these flow fields.

**Figure 5:**
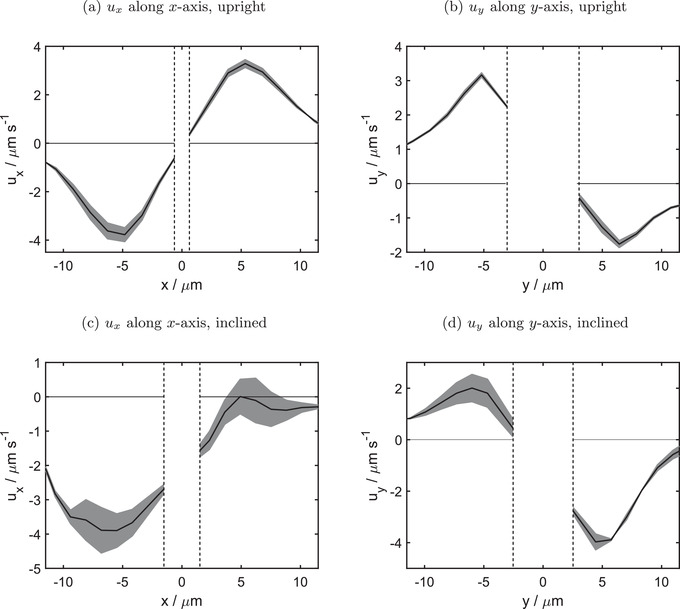
Velocities *u*_*x*_ along *x*‐axis and *u*_*y*_ along *y*‐axis of a,b) upright and c,d) inclined oriented pinned BiVO_4_ microswimmer. Dashed horizontal lines mark the borders of the pinned colloids. Black solid lines represent the average over two raster rows that intersect the particle center, upper and lower limits of grey colored area are the velocity values of these two rows. One raster row has a width of 1.1µm.

Flow fields of two exemplary individual BiVO_4_ microparticles can be found in **Figure** **4**a,b. For illustration, the original orientation of an upright and an inclined microswimmer have been added to the flow fields as yellow particle sketches. Additional flow fields can be found in Figure S6, Supporting Information. Comparison shows that tracer velocities and motion directions vary between the individual flow fields, especially in the far field. This observation is not surprising, as these microparticles are characterized by synthetic defects that render a unique profile of the spatial resolution of photocatalytic activity for each microswimmer. Additionally, the individual inclination angles toward the substrate influence the illumination of the particle facets. Yet, common trends can be determined. All of the analyzed microparticles exhibit inward flows from their oxidative ({110}) toward the reductive ({010}) facets, from where the fluid appears to be pushed outward. These results are in agreement with expectations based on the production and consumption sites of H^+^ ions (see Figure 2a,c). It becomes apparent that the source quadrupole, which is caused by opposite {010} facets (along the *x*‐axis) and opposite side faces with {110} facets (along the *y*‐axis) causes a flow field with similarities to a pusher‐type force dipole, which indicates that the flow direction caused by chemical reactions that produce and consume protons is overlaid by dominant hydrodynamic interactions. Similar observations have been obtained for fluid flows around immobilized induced‐charge electro‐osmotic (ICEO) Au@SiO_2_ janus particles.^[^ ^18^ ^]^ Due to symmetry axes of the particles and the applied electric field, a quadrupolar fluid flow field is indicated by theoretical considerations. Experimentally, this results in a flow pattern with force dipole similarities whose symmetry is broken by the different polarizability of the two materials.^[^ ^18^ ^]^ More detailed information about the flow profiles around the differently oriented microparticles can be obtained by analysis of velocities *u*_*x*_ and *u*_*y*_ along the *x*‐ and *y*‐axes of the rastered coordinate system, whose point of origin was placed in the center of the microparticles in each flow field. Therefore, analysis of *u*_*x*_ along the *x*‐axis enables comparison of flow velocities between the two reductive {010} facets. Equivalently, the difference between the oxidative {110} facets is characterized by *u*_*y*_ along the *y*‐axis. Results for the upright oriented particle are shown in **Figure** **5**a,b, and for the inclined particle in Figure 5c,d. The velocities *u*_*x*_ and *u*_*y*_ were calculated from the respective flow fields by averaging over two raster rows for *u*_*x*_ and columns for *u*_*y*_. The area covered by the microparticle is indicated by dashed lines in all plots. The tracer flow appears to be maximized at a distance of about 5 µm from the particle surface in all directions, independent of whether the microswimmers are attached in inclined or upright position and along which axis the velocity is analyzed. At shorter distances, we observed weak attractive interactions between the microswimmers and the tracers, which may be the reason for a decrease in their velocity. We also observe that few gold tracers attached to the microswimmers during flow field analysis, which is likely caused by van der Waals interactions. These tracer particles were then excluded from the flow field analysis. At higher distances than 5 µm, the impact of self‐induced flows by BiVO_4_ on the tracers decreases and their motion direction is randomized by Brownian motion, which leads to a decrease of *u*_*x*_ and *u*_*y*_ toward 0 µm s^−1^. However, differences in *u*_*x*_ and *u*_*y*_ between the upright and inclined particle are also observed. If *u*_*x*_ is compared between the two colloids, it can be seen that mirrored profiles are obtained for the two reductive sides of the upright particle (Figure 4a), with similar maximum velocities of *u*_*x*_ ≈ 4 µm s^−1^ . For the inclined particle however, this is not the case. While tracers are accelerated to similarly high velocities around 4 µm s^−1^ on its left side, which is oriented toward the glass substrate and therefore also the illumination source, lower values between 1 and −1 µm s^−1^ are obtained on the right side, which points away from the substrate (Figure 5c). Likely, self‐shadowing of the right particle side is responsible for the difference observed here. Velocity profiles of *u*_*y*_ along the *y*‐axis look similar for both particles, with differences of around 1 µm s^−1^ between the two oxidative sides of the colloids (Figure 5b,d). As excitation from the bottom illuminates these side faces to the same extent independent of the inclination angle, differences in velocity are likely caused by impacts of the unique shape of each particle on its catalytic activity.

In summary, we find that analysis of the tracer velocities supports the prediction, that the asymmetric chemical field around single crystalline BiVO_4_ microswimmers is caused by a combination of charge‐carrier separation and self‐shadowing. Note, that these analyses illustrate the flows around a 3D object in a 2D field, and neglect influences stemming from additional oxidative particle faces that are oriented toward the glass substrate and the top of the attached particles. Presumably, a similar trend of tracer flow from these oxidative to reductive particle faces is caused here, but further analysis would be necessary for confirmation. However, as the particles only perform motion within the *x*–*y*‐plane, analysis of 2D fields gives an overview of the important flows necessary for motion. Also, it should be noted that the flow fields discussed here are obtained from pinned microparticles. When the colloids are in motion, substrate‐related flows are expected to impact the flow field.^[^ ^17^ ^]^ Studies on the role of the substrates clearly indicate that the interactions are important for the swimming properties.^[^ ^44^, ^45^ ^]^ However, the experimental realization of particle tracking velocimetry benefits from the pinning of the active particles, since surface inhomogeneities and other imperfections that would lead to disturbance is suppressed. For experimental studies on spherical janus particle microswimmers (Pt@PS), flow fields with the same trend in flow direction, but smaller amplitudes have been obtained for moving microparticles compared to pinned ones.^[^ ^16^ ^]^ If a similar effect can be expected for the here investigated microswimmers, the flow fields do not only explain the presence of active motion itself, but also reflect the frequently observed tendency for circular trajectories. Enhanced fluid velocities with different amplitudes are caused along both, *x*‐ and *y*‐axis of the particle, which can be related to the persistent rotation which is especially observed for upright oriented particles. Inclined microswimmers, however, are often seen to move along rather straight trajectories, which may be caused by the absence of catalytic activity and therefore enhanced flow velocities along the self‐shadowed side of the particles.

**Figure 6:**
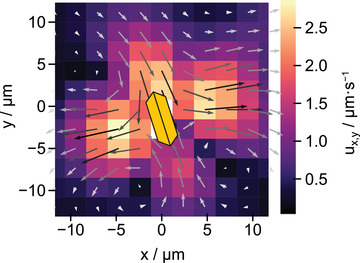
Mean flow field obtained by averaging over the flow patterns from four individual particles with an average size of 5 µm and an inclination angle of 90°. A common trend for flows from oxidative to reductive colloid sides is observed.

To facilitate the comparison between the different individual swimmers, we extract common trends in flow direction. To do so the flow fields of four individual BiVO_4_ colloids have been averaged. To ensure comparability, only data for particles with similar size (edge length of 5 µm) and upright orientation toward the substrate (α = 90°) have been considered. The obtained mean flow field is illustrated in **Figure** **6**. Here, it is clearly seen that the trend for the flow direction from oxidative to reductive sides is confirmed. Standard deviations of flow speed magnitude and direction of the mean flow field given in Figure S7, Supporting Information underline that, although different microswimmers cause varying magnitudes of flow speed due to individual synthetic surface defects, comparable flow directions exist. This illustrates, that charge carrier separation due to a surface heterojunction is indeed a sufficient source for an asymmetric chemical gradient around single crystalline BiVO_4_ microswimmers.

## 4 Conclusion

Within this manuscript we developed single‐component BiVO_4_ microswimmers with increased activity that rely on an inherent mechanism for charge carrier separation. Compared to conventional catalytic janus particle microswimmers, these colloids can be synthesized with a higher yield and are due to the cheap and nontoxic components ideal candidates for application‐related research in environmental remediation. Additionally, we recently also demonstrated that our microswimmers are not limited to H_2_O_2_ decomposition but can also be applied as catalysts in organic reactions relevant to the pharma industry. The clear separation of charge carriers onto different facets enabled us to predict a dominant self‐electrophoretic motion mechanism originating from a quadrupolar source field for the circular motion patterns observed under UV illumination in dilute H_2_O_2_ solutions. Particle tracking velocimetry of passive gold tracer particles allowed the illustration of flow fields around individual pinned BiVO_4_ colloids with different size and inclination angles and the confirmation of our conjectures on the motion mechanism. A common trend for inward flows toward oxidative facets and outward flows from reductive facets can be identified, which resembles the flow profile expected for a pusher. We observe the flows to be asymmetrized by individual surface defects and self‐shadowing, resulting in an overlay of the source quadrupole with hydrodynamic effects. Herein we demonstrated that the determination of flow fields can be a powerful tool to investigate and understand the source of colloid motion with various shapes. To our knowledge, this work represents the first realization of flow fields around both photocatalytic and nonspherical microswimmers. We visualized, that the illumination direction as well as the particle orientation contribute greatly to the interactions between the microswimmer surface and solute molecules. Self‐shadowing is a phenomenon relevant to all microswimmers driven by light‐absorption. Our findings are therefore not only relevant for truncated bipyramidal microswimmers but can pave the way to better understand other light‐driven phoretic systems. In future studies, the spatiotemporal resolution of flows around moving microswimmers, possibly even in 3D^[^ ^46^, ^47^, ^48^, ^49^ ^]^ as well as experiments of positively charged and neutral tracer particles will be attempted. These will extend the profound understanding of motion and interaction behavior, which is the key to design microswimmers for desired applications such as the investigation of collective behavior and cargo transport.

## 5 Experimental Section

### Synthesis of BiVO_4_ Microparticles

For the synthesis of single crystalline BiVO_4_ microparticles, a procedure by Xie et al. was adapted.^[^ ^35^ ^]^ In a 40 mL glass vial, 2.425 g Bi(NO_3_)_3_ · 5H_2_O and 0.585 g NH_4_VO_3_ were dissolved in 20 mL 2 m HNO_3_ under stirring and ultrasonication. After dissolution, the pH was adjusted to 2 with 25 wt% NH_3_. For shape control, NaCl with final concentrations of 0.05 and 0.2 m and sodium dodecyl sulfate with concentrations between 0.05 and 0.005 m were then added. After stirring for 15 min, the dispersion was transferred to a Teflon‐lined stainless steel autoclave and left to ripen for 2h. Subsequently, the synthesis was heated to 200 °C for 24 h. After cooldown to room temperature, the samples were washed with deionized water and ethanol extensively and dried under air at 70 °C overnight.

### Synthesis of Au Nanoparticles

Au nanoparticles were synthesized by a seeded growth method. For the seed synthesis, 500 µL 1 wt% HAuCl_4_ were diluted in 50 mL deionized water in a two‐necked 100 mL round bottom flask and heated to reflux. Subsequently, 2 mL 1 wt% sodium citrate were injected into the solution which was then refluxed for 5 min. After cooldown to room temperature, the seeds were stored under ambient conditions.

For seeded growth, a modified procedure from Liu et al. was followed.^[^ ^50^ ^]^ In a 10 mL glass vial, 4.8 mL deionized water and 25 µL 1 wt% sodium citrate were combined under stirring at 1000 rpm. Subsequently, 200 µL 1 wt% HAuCl_4_ were added, followed by 5 mL 30 wt% H_2_O_2_. Then, 5 µL Au nanoparticle seed solution were immediately injected and the solution was stirred for 5 min. For purification, the solution was centrifuged and the supernatant discarded. The obtained Au nanoparticles were dispersed in 1 mL deionized water and stored under ambient conditions.

### Powder XRD

XRD patterns were acquired using a Bruker 2D phaser in a 2θ range of 10–100°, where symmetrical scans were performed. The microparticles were dispersed in ethanol and drop‐casted onto a silicon wafer.

### SEM

For SEM imaging, dilute dispersions of BiVO_4_ microparticles in ethanol were drop‐casted onto silicon wafer pieces and attached to a specimen support with conductive carbon tape. Samples were dried overnight and imaged on a Zeiss Ultra Plus microscope with an acceleration voltage of 3 kV.

### Transmission Electron Microscopy (TEM)

For transmission electron microscopy (TEM) imaging, dilute dispersions of Au nanoparticles in ethanol were drop‐casted onto carbon‐coated copper TEM grids and dried under vacuum overnight. Images were taken on a FEI Tecnai F30 microscope (300kV).

### UV–Vis Spectroscopy

Absorption spectra were obtained on a Cary 600 spectrometer by placing a dilute dispersion of BiVO_4_ microparticles in deionized water in a quartz cuvette in an integrating sphere. Spectra were measured between 350 and 600 nm.

### Light Microscopy Experiments

For speed analysis, BiVO_4_ microparticles were dispersed in deionized water and combined with dilute H_2_O_2_ on cleaned 24 × 24mm glass substrates. Therefore, 9 µL particle dispersion was combined with 1µL of 1 wt% H_2_O_2_ solution, yielding a final concentration of 0.1 wt% H_2_O_2_. Analysis was performed in an inverted Zeiss microscope with a flexible Colibri 7 lightsource. Illumination with UV light (385 nm) was focused by a 63 × air objective to an intensity of 5.6 W cm^−2^. Videos were recorded with 40 fps.

For flow field experiments, a total volume of 10 µL of 0.2 wt% H_2_O_2_ with BiVO_4_ microswimmers and Au tracer particles was applied to a glass substrate and covered by a 18 × 18 mL cover slide. Subsequently, the samples were sealed with nail polish and inserted in the inverted Zeiss microscope. Videos were recorded under illumination with UV light that was focused by a 63 × air objective to 3.8 W cm^−2^ with a frame rate of 40 fps.

### Video Processing

For speed and mean‐squared displacement analysis of the microparticles, videos were converted to binary images and particles were tracked as Gaussian blobs in each video frame by the Trackmate plugin of the software ImageJ.^[^ ^51^ ^]^ For data processing, a MATLAB script based on the open‐source msdanalyzer class was used.^[^ ^52^ ^]^ Mean speeds were calculated as the unweighted mean of at least 20 individual particle speeds over a time range of 15 s from different video recordings. The speed is defined as the overall distance travelled by a particle in a given time.

### Flow Field Analysis

For obtaining the flow field near the immobilized microswimmers, the movement of intentionally added Au nanoparticles was recorded with the already mentioned widefield microscope. Localization of the tracer particles in individual frames as well as the linking of these positions to trajectories over successive images was conducted with the algorithm by Crocker and Gier^[^ ^53^ ^]^ using the TrackPy package. In order to remove spurious trajectories, only trajectories with a minimum length of ten frames were considered. Furthermore, erroneous detections inside the microswimmer and particles stuck to it were eliminated by discarding all particles inside a predefined polygon that covered approximately the area of the microswimmer. Subsequently, for every interrogation area in the grid the median of all displacement vectors was calculated and assigned to that position. For grid points without measurement data, the flow field was interpolated using a modified algorithm from the package PTVPy.

For merging the corresponding flow fields, the coordinate systems of these different measurements were transformed so that all microswimmers exhibited the same position and orientation. Next, the flow field was obtained via interpolation from all trajectories similar to the already described procedure. In order to ensure that all microswimmers contributed an equal amount to the result, the corresponding measurements were weighted inversely proportional to the number of recorded displacement vectors. For every interrogation area, the weighted mean was calculated as well as the corresponding standard deviation (see Supporting Information).

## Conflict of Interest

The authors declare no conflict of interest.

## Supporting information

Supporting Information

Supporting Information

Supporting Information

Supporting Information

Supplemental Video 1

Supplemental Video 2

## Data Availability

The data that support the findings of this study are available from the corresponding author upon reasonable request.
