# Supplementary material for: Beyond Janus Geometry: Characterization of Flow Fields around Nonspherical Photocatalytic Microswimmers
Source: Adv Sci (Weinh). 2022 Jul 15;9(24):2105009. doi: 10.1002/advs.202105009 (PMC9403636; doi:10.1002/advs.202105009)
Supplement: Supplementary file 1 — Supporting Information [file ADVS-9-2105009-s004.pdf]

ADVANCED SCIENCE
Open Access

Supporting Information

for *Adv. Sci.*, DOI 10.1002/advs.202105009

Beyond Janus Geometry: Characterization of Flow Fields around Nonspherical Photocatalytic Microswimmers

*Sandra Heckel, Clemens Bilsing, Martin Wittmann, Thomas Gemming, Lars Büttner, Jürgen Czarske and Juliane Simmchen**

Supporting Information: Beyond Janus Geometry: Characterization of Flow Fields Around Nonspherical Photocatalytic Microswimmers

*Sandra Heckel Clemens Bilsing Martin Wittmann Thomas Gemming Lars Büttner Jürgen Czarske Juliane Simmchen**

1 Overview of Supporting Videos

Table S1: Content and experimental conditions of supporting videos.

Video	Content	Conditions
S1	Motion of single crystalline $BiVO_4$ microswimmers	385 nm illumination ($5.6\,W\,cm^{-2}$), 0.1 wt% H_2O_2
S2	Motion of Au tracers around pinned microswimmer	385 nm illumination ($3.8\,W\,cm^{-2}$), 0.2 wt% H_2O_2

2 Characterization of $BiVO_4$ Microparticles

3 Identification of Electron- and Hole-Rich Facets Under Illumination

The location of charge carriers under illumination was identified by photodeposition of a metal (Ag) and a metal oxide (Co_3O_4). Therefore, a dilute dispersion of $BiVO_4$ colloids was combined with a solution of either $AgNO_3$ or $CoCl_2$ and $NaIO_3$ as an electron scavenger. Subsequently, the solutions were illuminated by a high power halogen lamp for (1...3) h and characterized by SEM. Results agree with first findings by Li *et al.*[1]: Silver is only reduced onto {010} facets, where electrons are located under illumination. Cobalt oxide however is only deposited onto {110} facets, which indicates that photogenerated holes are able to drive an oxidation reaction there.

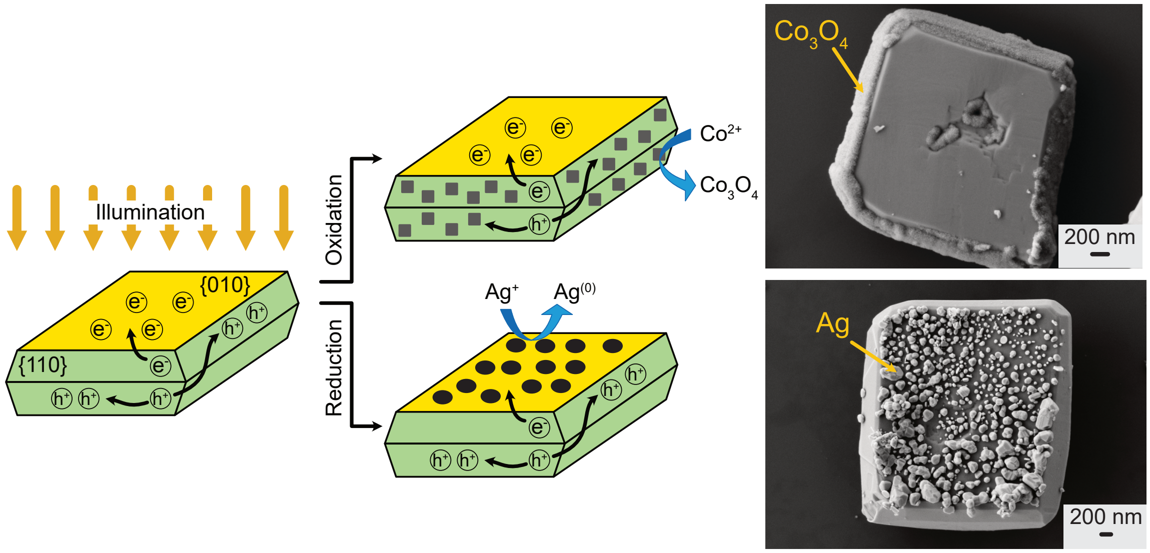

Figure S1: Photodeposition was applied as a means to identify the location of charge carriers under illumination. As can be seen in SEM images, Co_3O_4 is oxidized onto {110} facets, while Ag is reduced onto {010} facets.

4 Mean-Squared Displacement of Exemplaric $BiVO_4$ Microswimmer

Mean-squared displacement (MSD) of an exemplary single crystalline $BiVO_4$ microswimmer was calculated by a Matlab script based on the msdanalyzer class.[2] The circular motion pattern of the particles

is reflected in the MSD plot with linear axis graduation (**Figure S2a**), which has a periodic appearance. Brownian and active motion can clearly be distinguished by evaluating the MSD within $\tau \leq 1\,\mathrm{s}$, where directed motion can be expected.[3] In **Figure S2b**, this regime is shown in a bi-logarithmic plot. Indicators for $\propto \tau$ (Brownian motion) and $\propto \tau^2$ (upper boundary for directed motion) are also given. It can clearly be seen that the microswimmer performs active, directed motion.

(a) (b)

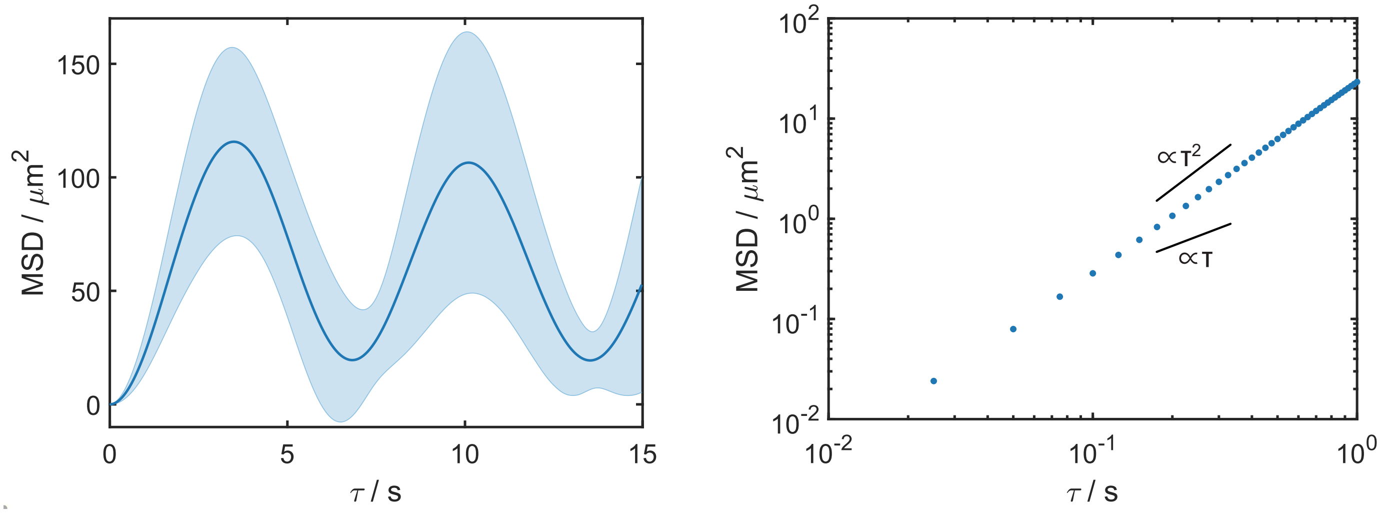

Figure S2: MSD of an exemplary microswimmer with (a) linear and (b) logarithmic axes. The trend for circular trajectories is reflected in the periodic MSD curve in (a), where the blue shaded area represents the standard deviation. The directed motion on timescales of $\tau \leq 1\,\mathrm{s}$ can be seen in (b). Indicators show $\propto \tau$ and $\propto \tau^2$.

5 Surface Defects of $BiVO_4$ Single Crystals

As can be seen in **Figure S3**, many of the single crystalline $BiVO_4$ colloids possess synthetic surface defects in the shape of fissures or scratches, which impacts the photocatalytic activity profile of each particle side and causes a unique flow profile despite the overall charge carrier separation mechanism (see Figure S1).

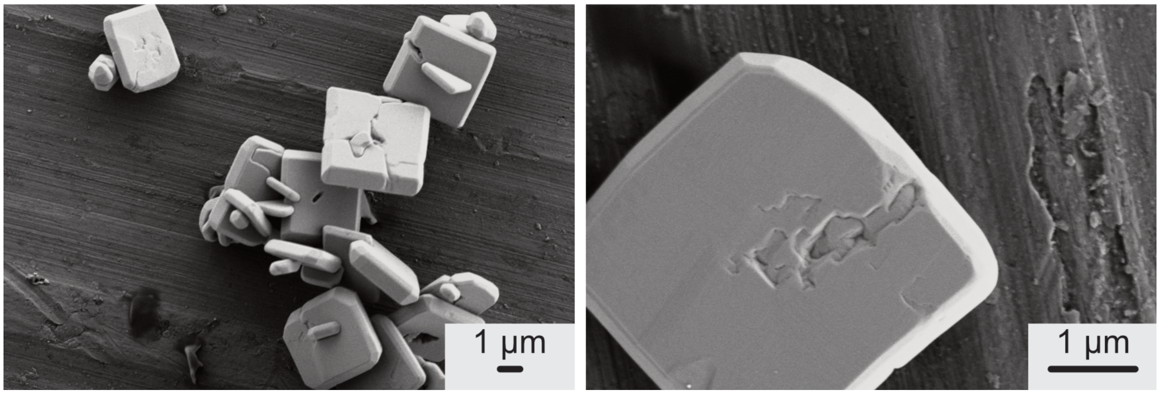

Figure S3: Surface scratches, defects and broken corners on the particles can be seen. These presumably cause facet-wise identical particle sides to possess different catalytic activities.

6 Self-Shadowing and light penetration depth

To assess the light transmission of a single particle and consequently the extent of self-shadowing, the penetration depth of UV light can be calculated from the absorption coefficient $\alpha_{385\,\mathrm{nm}} = 3 \times 10^{-4}\,\mathrm{cm}^{-1}$ of $BiVO_4$ in dependence of the semiconductor thickness δ_p.[4] The decrease of the light intensity I relative to the initial intensity I_0 can then be calculated as follows:

$$\frac{I}{I_0} = e^{-\alpha \cdot \delta_p} \tag{S1}$$

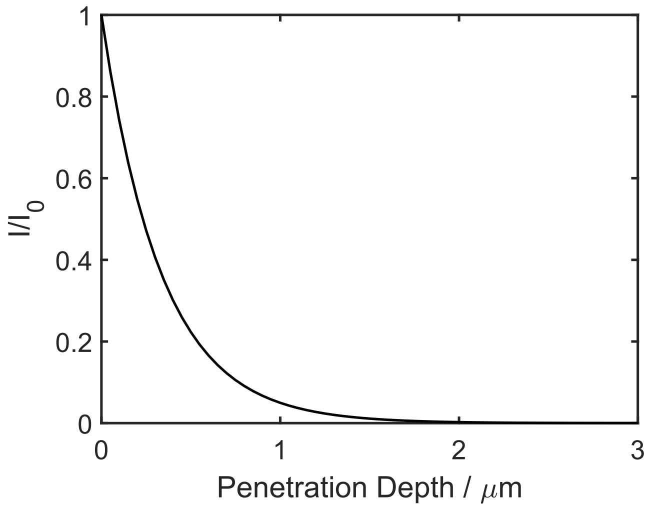

Figure S4: Decrease of the relative light intensity I/I_0 in dependence of the $BiVO_4$ thickness δ_p.

The light intensity ratio of $\frac{I}{I_0}$ versus the $BiVO_4$ particle thickness is displayed in **Figure S4**. It can be seen, that for the mean particle thickness of $(0.84 \pm 0.34)\,\mu\mathrm{m}$, the light intensity has decreased to $8\,\%$ of its initial value. Thus, an illuminated and a dark particle side form under illumination. These theoretical considerations are supported by the measurement of the absorption of a monolayer of polycrystalline $BiVO_4$ particles in a previous work.[5]

7 Au Nanoparticles

Gold nanoparticles were synthesized by a seeded growth approach of $HAuCl_4$ reduction by H_2O_2 in presence of citrate as a stabilizing ligand. The resulting particles have a quasispherical shape (see **Figure S5a**). Calculation of their mean diameter from TEM images yields a size of $(250 \pm 22)\,\mathrm{nm}$.

To assess the catalytic activity of the particles, a titration series was carried out. Therefore, three samples were prepared: A solution with only H_2O_2, a dispersion of Au nanoparticles in H_2O_2 and a dispersion of $BiVO_4$ microswimmers and Au nanoparticles in H_2O_2. Concentrations were equivalent to the flow field experiments. Each sample was illuminated with a UV LED for 15 min. Three aliquotes of 0.1 ml each were taken after 0 min, 5 min, 10 min and 15 min. The samples were diluted in a 1:1 mixture of H_2O and $2.5\,\mathrm{mol\,l^{-1}}$ H_2SO_4 and then titrated with $KMnO_4$ to determine the remaining H_2O_2 concentration (see **Equation S2** and **S3**).

$$5\,\mathrm{H_2O_2} + 2\,\mathrm{KMnO_4} + 4\,\mathrm{H_2SO_4} \rightarrow 2\,\mathrm{KHSO_4} + 2\,\mathrm{MnSO_4} + 8\,\mathrm{H_2O} + 5\,\mathrm{O_2} \tag{S2}$$

$$c_{\mathrm{H_2O_2}} = 2.5 \cdot \frac{c_{\mathrm{KMnO_4}} \cdot V_{\mathrm{KMnO_4}}}{V_{\mathrm{H_2O_2}}} \tag{S3}$$

The results are shown in **Figure S5b**, which shows the relative H_2O_2 concentration over time for all three samples. As can be seen, H_2O_2 is also decomposed to a minor extent without any particles present. This effect is known as the self-decomposition of H_2O_2 under UV illumination.[6] When Au nanoparticles were added, the amount of decomposed H_2O_2 decreased slightly. This shows, that the nanoparticles do not contribute to the H_2O_2 decomposition and therefore have no catalytic properties in this experiment. Instead, they absorb a fraction of the UV light, which is then unavailable for H_2O_2 decomposition. When $BiVO_4$ single crystals were added, the H_2O_2 is clearly decomposed under illumination, which confirms their photocatalytic activity as the dominant decomposition pathway of H_2O_2 under these conditions.

(a) (b)

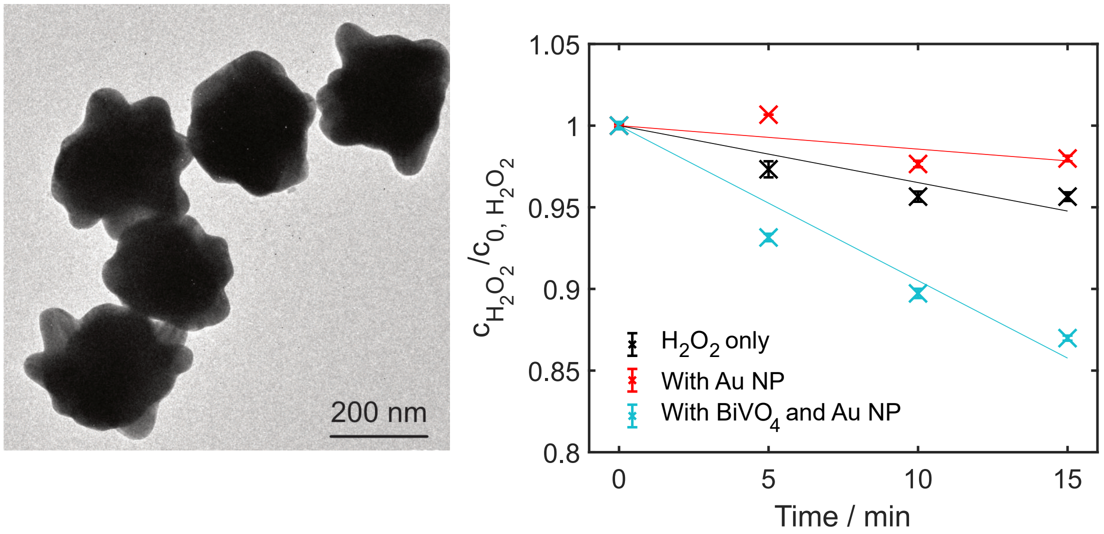

Figure S5: (a) TEM image of Au nanoparticles with quasi-spherical shape. (b) Titration of H_2O_2 with $KMnO_4$ in a solution of 0.2 wt% H_2O_2 only (black), a solution with additional Au nanoparticles (red) and a solution with additional Au nanoparticles and $BiVO_4$ microparticles (cyan).

8 Additional Flow Fields

Additional flow fields around pinned single crystalline $BiVO_4$ microparticles are shown in **Figure S6** in the laboratory frame of reference. In all examples, the size and orientation of the original colloids are replicated by the yellow particle sketches. Although flow fields vary between individual microparticles, common trends of inward flows to the {110} facets and outward flows from the {010} facets can be observed.

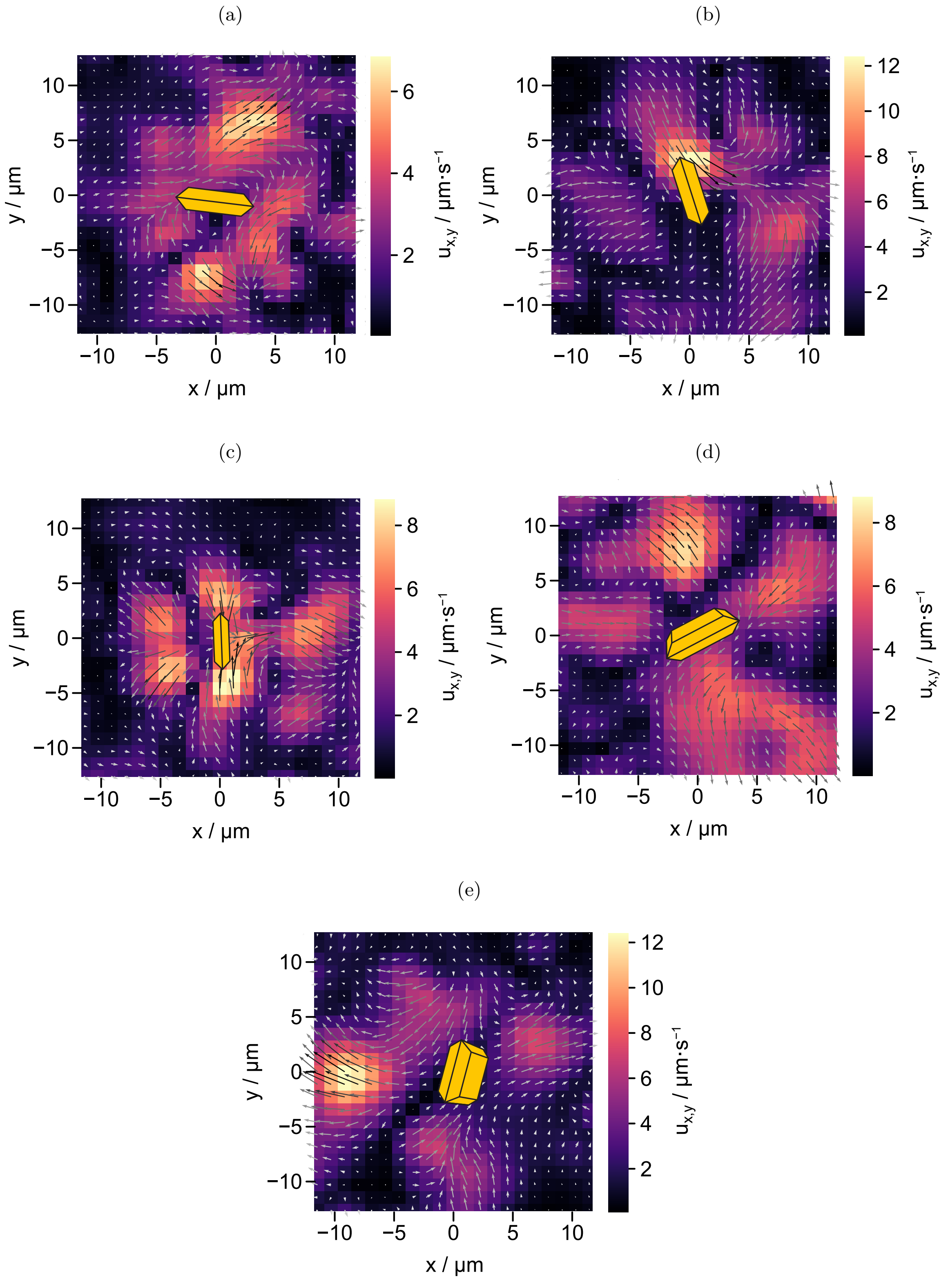

Figure S6: Additional flow fields of pinned individual $BiVO_4$ microparticles. The position and orientation of the colloids is indicated by schematic sketches.

9 Standard Deviation of Mean Flow Field

The standard deviation σ of the mean flow field determined by averaging over four individual flow fields was calculated for the flow direction and the flow speed separately and is shown in **Figure S7**. The stan-

dard deviation of the flow direction was determined as σ_ϕ for the angle ϕ of a polar coordinate system in each raster point (Figure S7a). If the gold tracers only show Brownian behavior, all motion directions have the same probability. Their motion direction can then be described by a continuous uniform distribution which means that a threshold of $\sigma_{\phi,t} = 104°$ can be calculated (**Equation S4**):[7]

$$\sigma_{\phi,t} = \frac{\phi_{max} - \phi_{min}}{2\sqrt{3}} = \frac{360° - 0°}{2\sqrt{3}} = 104° \tag{S4}$$

A value around 104° is observed at distances greater than 10 µm from the microswimmer surface in all directions. In the area within 10 µm from the microswimmer surface, lower values of σ_ϕ indicate a preferred flow direction. Secondly, the standard deviation of the speed magnitude was determined as the relative value $\sigma_{v_{rel}}$ (Figure S7b). It can be seen that the standard deviation is highest close to the microswimmer surface, which indicates that tracer particle speeds are influenced to a different extent by the different individual microswimmers chosen for averaging. For differences greater than 5 µm from the microswimmer surface in all directions, the impact of the self-induced flows by the $BiVO_4$ colloids on the fluid flow decreases, which also leads to a decrease in relative standard deviation.

(a) (b)

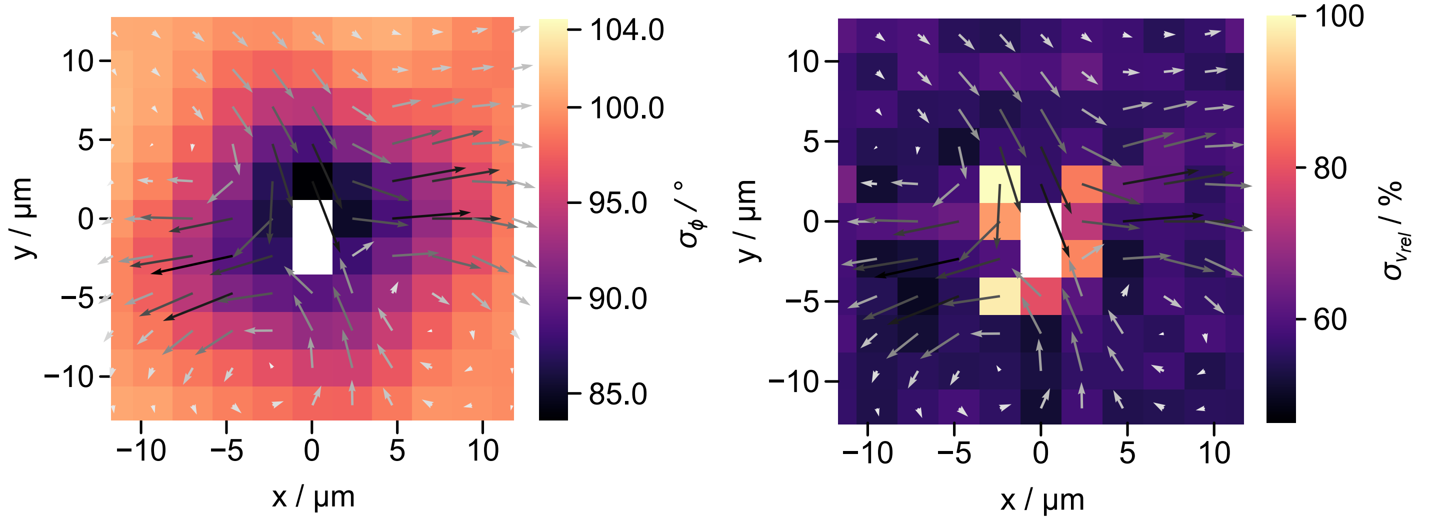

Figure S7: (a) Standard deviation σ_ϕ of the flow direction angle ϕ in a polar coordinate system and (b) standard deviation of the flow speed magnitude $\sigma_{v_{rel}}$ of the relative speed v_{rel}.

References

[1] R. Li, F. Zhang, D. Wang, J. Yang, M. Li, J. Zhu, X. Zhou, H. Han, C. Li, *Nature Communications* **2013**, *4* 1432.

[2] N. Tarantino, J. Y. Tinevez, E. F. Crowell, B. Boisson, R. Henriques, M. Mhlanga, F. Agou, A. Israël, E. Laplantine, *Journal of Cell Biology* **2014**, *204*, 2 231.

[3] D. Yamamoto, A. Shioi, *KONA Powder and Particle Journal* **2015**, *32* 2.

[4] Z. Zhao, Z. Li, Z. Zou, *Physical Chemistry Chemical Physics* **2011**, *13*, 10 4746.

[5] S. Heckel, Ph.D. thesis, Technische Universität Dresden, **2021**, URL `https://nbn-resolving.org/urn:nbn:de:bsz:14-qucosa2-769616`.

[6] E. Abel, *Monatshefte für Chemie* **1952**, *83*, 2 422.

[7] C. Hesse, *Angewandte Wahrscheinlichkeitstheorie*, Vieweg+Teubner Verlag, Wiesbaden, 1 edition, **2003**.
